# Antimicrobial peptide 2K4L disrupts the membrane of multidrug-resistant *Acinetobacter baumannii* and protects mice against sepsis

**DOI:** 10.3389/fmicb.2023.1258469

**Published:** 2023-10-24

**Authors:** Fangyu Ji, Guoxu Tian, Dejing Shang, Fengquan Jiang

**Affiliations:** ^1^School of Life Science, Liaoning Normal University, Dalian, China; ^2^Liaoning Provincial Key Laboratory of Biotechnology and Drug Discovery, Liaoning Normal University, Dalian, China; ^3^Department of Clinical Laboratory, The First Affiliated Hospital of Dalian Medical University, Dalian, China

**Keywords:** antimicrobial peptide, multidrug resistance, *Acinetobacter baumannii*, membrane, biofilm, sepsis, inflammation

## Abstract

Antimicrobial peptides represent a promising therapeutic alternative for the treatment of antibiotic-resistant bacterial infections. 2K4L is a rationally-designed analog of a short peptide temporin-1CEc, a natural peptide isolated and purified from the skin secretions of the Chinese brown frog *Rana chensinensis* by substituting amino acid residues. 2K4L adopt an α-helical confirm in a membrane-mimetic environment and displayed an improved and broad-spectrum antibacterial activity against sensitive and multidrug-resistant Gram-negative and Gram-positive bacterial strains. Here, the action mechanism of 2K4L on multidrug resistant *Acinetobacter baumannii* (MRAB) and protection on MRAB-infected mice was investigated. The results demonstrated high bactericidal activity of 2K4L against both a multidrug resistant *A. baumannii* 0227 strain (MRAB 0227) and a sensitive *A. baumannii* strain (AB 22934), indicating a potential therapeutic advantage of this peptide. Strong positively-charged residues significantly promoted the electrostatic interaction on 2K4L with lipopolysaccharides (LPS) of the bacterial outer membrane. High hydrophobicity and an α-helical confirm endowed 2K4L remarkably increase the permeability of *A. baumannii* cytoplasmic membrane by depolarization of membrane potential and disruption of membrane integration, as well as leakage of fluorescein from the liposomes. Additionally, 2K4L at low concentrations inhibited biofilm formation and degraded mature 1-day-old MRAB 0227 biofilms by reducing the expression of biofilm-related genes. In an invasive *A. baumannii* infection model, 2K4L enhanced the survival of sepsis mice and decreased the production of the proinflammatory cytokines downregulating the phosphorylation level of signaling protein in MAPK and NF-κB signaling pathways, indicating that 2K4L represents a novel therapeutic antibiotic candidate against invasive multidrug-resistant bacterial strain infections.

## 1. Introduction

Microbial resistance to antibiotics has proven to be a serious public health problem that urgently needs to be addressed. The emergence and prevalence of multidrug resistant (MDR) pathogenic bacteria poses a serious threat to human health worldwide (Krishnamurthy et al., 2016). Antibiotic-resistant infections kill approximately 5.3 million people each year globally. If effective strategies do not counteract these hospital-acquired infections, an estimated 10 million deaths annually will be caused by the infections by 2050 (Avershina et al., 2021). *Acinetobacter baumannii* is one of the most multidrug-resistant pathogens of the ESKAPE group that causes hospital-acquired infections (*Enterococcus faecium*, *Staphylococcus aureus*, *Klebsiella pneumoniae*, *A. baumannii*, *Pseudomonas aeruginosa*, and *Enterobacter* spp.) (Kumar et al., 2021; Dehbanipour and Ghalavand, 2022). *A. baumannii* can escape the attack of antibiotics and survive in a hospital environment for prolonged time periods because of its remarkable ability to take up genetic material encoding drug resistance from the environment, resulting in the emergence of multidrug resistance (Ayoub Moubareck and Hammoudi Halat, 2020). In addition, biofilms of *A. baumannii* act as a barrier for bacteria to resist external attacks, thus increasing bacterial resistance (Mea et al., 2021). Numerous clinical studies have shown that multidrug resistance of *A. baumannii* to the class antibiotics carbapenems, tigecycline, and colistin is now widespread, and carbapenem-resistant *A. baumannii* is ranked 1st in the list.

*A. baumannii* infections trigger excessive inflammatory reactions, leading to endotoxic shock, sepsis, and even death (Routsi et al., 2010; Muzaheed et al., 2017). Sepsis, is a systemic inflammatory response syndrome (SIRS) caused by infections of pathogenic microorganisms, especially gram-negative bacteria (Carcillo and Fields, 2002; Uhle et al., 2015; Sehgal et al., 2020). Throughout the course of sepsis, the massive release of proinflammatory factors caused by an excessive inflammatory response plays the most important role. Therefore, inhibition of an excessive inflammatory response is particularly important in the treatment of sepsis (Jacobi, 2002). Sepsis requires immediate treatment with intravenous fluids and antibiotics, and broad-spectrum antibiotics are recommended for severe sepsis (Dellinger et al., 2013; Rhee et al., 2020; Im et al., 2022). However, multidrug resistance among various bacterial strains is currently leading to worldwide resistance to a wide range of antibiotics. The overuse and misuse of antibiotics has led to the emergence of extensively multidrug-resistant bacterial strains resulting in increased mortality in patients with bacteremia or sepsis (Magrone et al., 2018; Luna-Reyes et al., 2021). Therefore, the development of new therapeutic antibiotics is urgently needed to combat *A. baumannii* infections and the side effects caused by *A. baumannii*. Among the various antimicrobial options, antimicrobial peptides (AMPs) have been widely regarded as a promising solution to combat MDR bacteria (Moravej et al., 2018). Unlike the bactericidal effects of traditional antibiotics, AMPs interact with bacterial membranes through electrostatic charges or hydrophobic groups and physically disrupt bacterial morphology (Mookherjee et al., 2020). This antimicrobial mechanism of membrane destabilization might be the most promising way to solve the multi-drug resistance problem because a fundamental changes in membrane composition be a very slow process (Moravej et al., 2018). In addition, it has been reported that AMPs not only have antimicrobial activity but also have antibiofilm, anti-inflammatory, and antitumor activity and could be potential therapeutic candidates for sepsis due to their multiple properties (Kumagai et al., 2020; Nagaoka et al., 2020).

The AMP 2K4L and 2K2L used in this study was designed and synthesized based on the amino acid sequence and structure of temporin-1CEc, a natural peptide from the skin secretion of *Rana chensinensis*, by substituting amino acid residues (Ji et al., 2022). Both peptides with 13 amino residues and amidation of carboxyl terminals adopt an α-helical secondary structure in a membrane-like environment. 2K4L and 2K2L showed broad-spectrum antibacterial activity against the sensitive and multidrug resistant bacteria. While 2K4L with the high hydrophobic value exhibited fourfold higher antibacterial activity than 2K2L although both peptides composed of the same net positive charges (+5). In the present study, the antibacterial activity and action mechanism of 2K4L against clinically isolated multidrug-resistant *A. baumannii in vivo* and *in vitro* were investigated. We aimed to elucidate the protective action of 2K4L on *A. baumannii*-induced sepsis mice and provide a potential therapeutic option for antibiotic-resistant pathogens.

## 2. Materials and methods

### 2.1. Peptides

The peptides (purity ≥ 95%) 2K2L (IIPLPLKKFLKKL-NH_2_) and 2K4L (IILLLLKKFLKKL-NH_2_) used in this study were synthesized by GL Biochemistry Inc (Shanghai, China). The results of reversed-phase high performance liquid chromatography (RP-HPLC) and MALDI-TOF mass spectrometry were shown in Supplementary Figure 1. Both 2K2L and 2K4L have net charges of +5, and the mean hydrophobicity is 14.94 and 17.14, respectively. Mean hydrophobicity values (H) for peptides were calculated according to Mant et al. (2009).

### 2.2. Bacteria

The multidrug-resistant *Acinetobacter baumannii* (MRAB 0227) strain was obtained from The First Affiliated Hospital of Dalian Medical University, and identification and antimicrobial susceptibility testing of the multidrug-resistant bacteria was performed by an automated MicroScan^®^ WalkAway 96 Plus system (Siemens Ltd., Germany). Standard strain *A. baumannii* CICC 22933 (AB 22933) was acquired from the China Centre for Industrial Culture Collection. Bacteria were grown in Luria Bertani (LB) medium at 37°C overnight.

### 2.3. Animals and ethical statement

SPF C57BL/6 male mice (6–8 weeks, 18–22 g) were purchased from Liaoning Changsheng Biotechnology Co., Ltd., and housed under standard conditions of light and temperature in the Laboratory Animal Center at Liaoning Normal University. Mice had free access to standard laboratory chow and water. All animal experiments in this study were approved by the Animal Use and Care Committee of Liaoning Normal University (the ethical approval number: LL2023062).

### 2.4. Minimum inhibitory concentration (MIC) assay

The minimum inhibitory concentration (MIC) of the peptides was determined using a standard microdilution method in 96-well microtiter plates. Briefly, bacterial cells were grown in LB medium at 37°C overnight. Then, 100 μL of diluted bacterial cultures (diluted to 2 × 10^5^ CFU/mL with LB medium) was mixed with 100 μL of serially diluted peptide solutions (concentration ranging from 3.13 to 200 μM) in a 96-well plate and incubated at 37°C for 16–18 h. The absorbance (OD_600_) of each sample was measured using a microtiter plate reader (Varioskan Flash Microplate Reader, Thermo Scientific Co., Beijing, China). The MIC was defined as the lowest peptide concentration that inhibited bacterial growth by 95%. Three duplicates were performed for each condition.

### 2.5. Time-kill kinetics assay

The bactericidal kinetics of peptides against *A. baumannii* were determined by time-kill curves. Briefly, log-phase cultures of AB 22933 and MRAB 0227 (1 × 10^5^ CFU/mL) were mixed with 10 μL of peptides at final concentrations of 0.5 × MIC, 2 × MIC and 8 × MIC and incubated with shaking at 37°C for 0–180 min. An incubation in the absence of peptide was used as a control. At 0, 15, 30, 60, 90, 120 and 180 min of incubation, the peptides were removed by centrifugation at 4,000 rpm for 10 min, and the serially diluted bacterial suspension was spread on agar plates and cultured at 37°C overnight. The number of bacterial colonies was calculated by linear regression analysis of a plot of log_10_ CFU versus time. There was no bacterial colony on the agar plates coated with the original bacterial solution, and the number of bacterial colonies was calculated as 0. Three duplicates were performed for each condition.

### 2.6. Scanning electron microscopy

The cell morphology of MRAB 0227 was observed by using scanning electron microscopy (SEM). Mid-log phase bacterial cultures (1 × 10^5^ CFU/mL) were treated with 1 × MIC and 2 × MIC of peptides at 37°C for 1 h. After washing 3 times with distilled water, bacterial cells were fixed with 2.5% glutaraldehyde (pH 7.2–7.4) for 2 h and then dehydrated by using a stepwise gradient of ethanol. The bacterial samples were smeared and spread on a glass slide, dried at room temperature and coated with approximately 5 ng of gold/palladium. The images were observed with a scanning electron microscopy (Hitachi SU8010, Hitachi, Japan).

### 2.7. Atomic force microscopy

The cell morphology of MRAB 0227 was further observed by using atomic force microscopy (AFM). Briefly, mid-log phase bacterial cultures (1 × 10^5^ CFU/mL) were treated with 0.5 × MIC and 2 × MIC of peptides at 37°C for 1 h. After washing 3 times with distilled water, bacterial cells were fixed with 2.5% glutaraldehyde (pH 7.2–7.4) for 2 h. Subsequently, 20 μL bacterial suspension was incubated on the mica for 30 min at room temperature. The alterations in the bacterial cell surface were imaged by MFP-3D Origin™ Atomic Force Microscope (Oxford Instruments, USA) in the contact operation mode with a spring constant of 0.08 N/m. Images of the topography and 3D-image were acquired simultaneously. The roughness values were measured over the entire bacterial cell surface on 200 nm × 200 nm areas. The average surface root-mean-square (RMS) roughness was calculated from ten fields of three cells estimated during two independent experiments. The data were analyzed with Asylum Research AFM (Santa Barbara, CA, USA).

### 2.8. Bactericidal activity assay

The bactericidal activity of peptides was examined using a LIVE/DEAD BacLight™ Bacterial Viability kit L7012 (Molecular Probes Inc., Eugene, OR, USA) according to the manufacturers’ instructions by using confocal laser scanning microscopy (LSM-710, Carl Zeiss Microimaging, Germany). Briefly, mid-log phase AB 22933 and MRAB 0227 (1 × 10^5^ CFU/mL) were treated with peptides at a final concentration of 0.5 × MIC and 2 × MIC at 37°C for 1 h. Polymyxin B (PMB) was used as a positive control. After washing three times with PBS, the bacterial cells were stained with the fluorescent dyes SYTO-9 and propidium iodide (PI) for 30 min at 4°C in the dark according to the kit instructions. The cells were washed three times with PBS, coated on a glass slide and fixed. The images were observed by confocal laser scanning microscopy, and SYTO-9 (green) and PI (red) fluorescence intensities were measured at wavelengths of 485 nm/530 nm and 485 nm/630 nm (excitation/emission), respectively. The bacterial live percentage was calculated by dividing the green fluorescence intensity by the red fluorescence intensity.

### 2.9. Fluorescein release assay

Large unilamellar vesicles (LUVs) composed of POPE/POPG/cholesterol were obtained to mimic the cell membranes of gram-negative bacteria as previously described (Konate et al., 2020). Briefly, POPE, POPG and cholesterol were dissolved in chloroform according to mass ratios of 1.85:0.15:1, 1:1:1 and 0.15:1.85:1, respectively, mixed and sonicated in an ultrasonic washer and then dried via vacuum evaporation to obtain a thin film on the wall of a round-bottomed flask. After the lipid film was hydrated with 5 mM TES buffer (pH 7.4), a calcein solution (dissolved in 50 mM TES, 100 mM NaCl, pH 7.4) was added to the sample. The calcein-loaded LUVs were prepared by ultrasonication over ten freeze-thaw cycles in liquid nitrogen until homogeneous and then extruded through 0.22-μm polycarbonate membranes. Free calcein was removed by gel filtration through a Sephadex G-50 column and eluted using TES buffer, and the LUVs encapsulating calcein were eluted with the void volume. Different concentrations of peptides (final concentration of 3.13–100 μM) were added to the calcein-loaded LUVs and incubated at 25°C for 30 min in the dark. PMB and piperacillin were used as positive and negative controls, respectively. The calcein fluorescence intensity was recorded at excitation/emission wavelengths of 480 nm/530 nm, and the calcein release rate was calculated by the following formula: calcein release rate (%) = 100 × (F-F_0_)/(Ft-F_0_), where F and Ft are the fluorescence intensity measured after adding peptides or Triton X-100, respectively, and F_0_ is the fluorescence intensity of PBS.

The preparation of CF and FITC-dextran type dye-loaded LUVs was identical to that of calcein-loaded LUVs. A mass ratio of POPE, POPG and cardiolipin was 18:4:3, which mimic the cell membranes of *A. baumannii* (Jiang et al., 2020). Three duplicates were performed for each condition.

### 2.10. Inner membrane depolarization

Bacterial membrane depolarization was measured using the membrane potential-sensitive fluorescent dye DiSC3-5 (Sigma Aldrich, Shanghai, China). Mid-log phase bacterial cultures were collected by centrifugation at 4,000 rpm for 10 min washed twice with 5 mM HEPES (pH 7.2), and resuspended in HEPES to 1 × 10^5^ CFU/mL. After the addition of 0.5 μM EDTA, the bacterial suspension was incubated with DiSC3-5 at a final concentration of 4 μM in a black NBS microplate until DiSC3-5 dye was taken up at a maximum amount by bacterial cells. Peptides at final concentrations of 1, 2 and 4 × MIC were added to the bacterial samples. Triton X-100 (0.1%) was used as a positive control. DiSC3-5 fluorescence was monitored at an excitation wavelength of 622 nm and an emission wavelength of 670 nm at intervals of 1 min. Membrane depolarization ability was examined by the dye fluorescence intensity change as recorded on a microtiter plate reader. For each dye concentration, at least three measurements were repeated. Three duplicates were performed for each condition.

### 2.11. NBD fluorescence measurements

NBD [N-(7-Nitrobenz-2-Oxa-1,3-Diazol-4-yl)-1,2-Dihexadecanoyl,MedChemExpress, USA]-labeled peptides (final concentration of 3.13–100 μM) were added to a black NBS microplate (Sun et al., 2015). LUVs (PE, PG and CL at a mass ratio of 18:4:3) were added to the microplate and the molar ratio of lipid to peptide was 1, 5, 10, 20, 40 and 60. After 65 min, proteinase K (10 μg/mL) was added. The fluorescence intensity was recorded every 5 min before and after the addition of proteinase K at the excitation and emission wavelengths of 470 nm and 530 nm, respectively. Three duplicates were performed for each condition.

### 2.12. Super-resolution fluorescence microscopy

Bacterial membrane images were observed by super-resolution fluorescence microscopy. Mid-log phase MRAB 0227 (1 × 10^5^ CFU/mL) was treated with the AF488 (Alexa Fluor 488, Invitrogen, Shanghai, China)-labeled peptide at a final concentration of 0.5 × MIC and 2 × MIC at 37°C for 1 h. After centrifugation at 4,000 rpm/min for 10 min, the bacterial cells were incubated with 20 μL FM™ 4-64FX dye (fixable analog of FM™ 4-64 membrane stain) at a concentration of 5 μg/mL on ice for 10 min with regular mixing. The cells were collected by centrifugation, washed with HBSS, and then fixed in 2% paraformaldehyde for 10 min at room temperature. Then, 3D-SIM was performed using a microscope system (DeltaVision OMX version; GE Healthcare, UK).

### 2.13. Outer membrane permeability

Outer membrane permeability was analyzed by using 1-N-phenylnaphthylamine (NPN, Solarbio, Beijing, China) dye (Sun et al., 2015). Ninety microliters of mid-log phase bacteria (1 × 10^5^ CFU/mL) was plated in a 96-well plate and mixed with 20 μL of 10 μM NPN solution and 10 μL of peptides at a concentration range from 3.13 to 200 μM. After incubating for 1 h, the fluorescence intensities of the samples were measured with an LB970 fluorescence microplate reader. The excitation and emission wavelengths were 350 nm and 420 nm, respectively. Gentamicin was used as a positive control. The untreated group was used as a negative control. The fluorescent intensity was calculated by the following formula: fluorescent intensity = F_p_-F_0_, where F_p_ and F_0_ are the fluorescence intensity measured after adding peptides or the negative control, respectively. Three duplicates were performed for each condition.

### 2.14. Zeta potential measurement

The zeta potentials of the bacterial outer membrane were measured on a Zetasizer Nano ZS (Malvern Instruments, UK). Mid-log phase bacteria (1 × 10^5^ CFU/mL) in disposable zeta cells were titrated with the peptides (concentration range from 1.56 to 50 μM). The determination of zeta potential was performed by a total of 5 measurements of 100 runs for all concentrations of the peptides. Three duplicates were performed for each condition.

### 2.15. LPS neutralization

Neutralization of lipopolysaccharides (LPS) was measured by using a pierce™ chromogenic endotoxin quant kit (Thermo Scientific, USA) according to the manufacturers’ instructions. The peptides were mixed with 1 EU/mL LPS working solution in pyrotube depyrogenated test tubes and incubated at 37 °C for 1 h. Then 100 μL of the mixture was added to an equal volume of limulus amebocyte lysate (LAL) reagent and was incubated at 37°C for 15 min. A total of 100 μL of the chromogenic substrate was added and incubated for 10 min, followed by the addition of 500 μL of azo reagent 1, 2, and 3 in sequence. After mixing, the mixture was added into a non-pyrogenic 96-well tissue culture plate and the yellow color that developed due to cleavage of the substrate was then measured at 545 nm. The neutralization proportional was calculated according to the standard curve, and the light absorption value of LPS working fluid of 1 EU/mL was set as a negative control. Three duplicates were performed for each condition.

### 2.16. Disassociation of LPS

FITC- LPS (SigmaeAldrich, Shanghai, China) was vortexed in 10 mM SP buffer (pH = 6.8) to a final concentration of 1 mg/mL. FITC- LPS aggregates were treated with peptides and the molar ratio of peptides to LPS was 0, 1, 2, 5, 10, 15 and 20. The fluorescence intensity was monitored with an emission wavelength at 515 nm and an excitation wavelength at 488 nm by using Varioskan Flash Microplate Reader (Thermo Scientific Co., Beijing). The molar ratio of peptides to LPS was 0 as a negative control. The fluorescent intensity was calculated by the following formula: fluorescent intensity = F_p_-F_0_, where F_p_ and F_0_ are the fluorescence intensity measured after adding peptides or the negative control, respectively. Three duplicates were performed for each condition.

Dynamic light-scattering (DLS) was used to measure the particle size distributions of LPS with and without peptides by Malvern Zetasizer Nano 2S90 (Malvern Instruments, UK). LPS solution in SP buffer (1 mg/mL) was heated to 60 °C and cooled to 20 °C repeatedly three times and then placed in 4 °C overnight to form LPS aggregates. The peptides were dissolved in SP buffer at a concentration of 1 mg/mL. Measurements were performed of LPS aggregates, the peptides and LPS with peptides after incubation for 30 min. The standard CONTIN method was used to analyze the data. Three duplicates were performed for each condition.

### 2.17. Preparation of bacterial protoplasts

Protoplasts were prepared according to previously described procedures (Figueroa et al., 2018). Briefly, mid-log phase bacteria were collected by centrifugation, washed twice with 10 mM phosphate buffer (pH 7.0), and then resuspended in 0.5 M sucrose solution in phosphate buffer at a final concentration of 1 × 10^7^ CFU/mL. Lysozyme (final concentration 80 μg/mL) and 0.5 μM EDTA were added to the bacterial suspension and incubated for 2 h at 37°C in a shaker. The sphere-shaped bacteria observed by an optical microscope showed the successful preparation of protoplasts. The solutions were washed twice with 0.25 M sucrose solution before resuspension in buffer containing 5 mM HEPES, 20 mM glucose and 100 mM KCl, pH 7.0. The MIC value of peptides against bacterial protoplast was determined according to the experimental method of MIC assay. Three duplicates were performed for each condition.

### 2.18. Biofilm formation assay

Biofilm formation of the MRAB 0227 and AB 22933 strains was examined according to our previous work (Shang et al., 2017). Briefly, 100 μL of mid-log phase bacterial cells (1 × 10^5^ CFU/mL) and 100 μL 1/8 × MIC, 1/4 × MIC, and 1 × MIC peptide were placed into a 96-well polystyrene microtiter plate and incubated at 37°C for 24 h. The suspended bacteria were carefully removed by centrifugation, and the adherent bacteria with biofilms were fixed with methanol for 15 min. The adherent bacteria with biofilms were resuspended and the viability of biofilm-associated cells was evaluated by CFU counting. Then the bacteria were stained with 0.1% (w/v) crystal violet dye for 5 min, resuspended with PBS and quantified by measurement at 590 nm in a microtiter plate reader (Varioskan Flash Microplate Reader, Thermo Scientific Co., Beijing, China). PMB and piperacillin (PIP) were used as positive and negative controls, respectively. The inhibition percentage of biofilm formation was calculated using the following formula: (ODnontreated–ODpeptide/ODnontreated) × 100%. The lowest concentration of peptide required to inhibit biofilm formation by ≥ 50% was defined as the minimum biofilm inhibitory concentration (MBIC_50_). Three duplicates were performed for each condition.

### 2.19. One-day-old biofilm disaggregation assay

Disaggregation of 1-day-old biofilms was determined as described by Shang et al. (2014). The formed 1-day-old biofilms were washed with PBS and then incubated with the peptides at concentrations of 1 × MIC, 2 × MIC, and 4 × MIC for 24 h. The biofilm was then fixed, stained, resuspended and quantified as described above. The minimum biofilm reduction concentration (MBRC_50_) was defined as the lowest peptide concentration that reduced the biofilm by ≥ 50%. Three duplicates were performed for each condition.

### 2.20. Quantitative real-time PCR

Mid-log phase MRAB 0227 cultures (final concentration of 1 × 10^5^ CFU/mL) were incubated with the peptides at concentrations of 1/4 × MIC, 1/2 × MIC, and 1 × MIC at 37°C for 1 h. After centrifugation and collection, RNA was extracted when the bacterial count reached 10^8^ CFU. The bacterial cultures were washed with PBS and digested with 20 μL lysozyme (3 mg/mL) after centrifugation at 10,000 rpm for 5 min. Then, preparation and reverse transcription of total RNA was carried out as described previously (Licht et al., 2005). cDNA was amplified with a HiScript^®^ II Q RT SuperMix for qPCR (+gDNA wiper) kit (Vazyme, Nanjing, China). Real-time quantitative PCR was performed with TB Green^®^ Premix Ex Taq™ II (Tli RNaseH Plus) (TaKaRa, Dalian, China) on an ABI-Prism 7500 Fast sequence detection system (Applied Biosystems). Gene expression levels were calculated according to the comparative cycle threshold (Ct) method after normalization with an internal control 16S rRNA. All primer sequences are shown in Table 1. The sequences were synthesized by Sangon Biotech (Shanghai, China). Three duplicates were performed for each condition.

**TABLE 1:** Genes and primer sequences.

Gene	Forward	Reverse	Gene ID
*bmfR*	CTGGTAGGTAATGCAGTTCG	GAGAGACCCAAACCATAACC	66398311
*ompA*	CTCTTGCTGGCTTAAACGTA	GCAATTTCTGGCTTGTATTG	66397663
*csuA/B*	ATGCGGTAAATACTCAAGCA	TCACAGAAATATTGCCACCT	43164957
*csuE*	ATGCATGTTCTCTGGACTGATGTTGAC	CGACTTGTACCGTGACCGTATCTTGATAAG	66396671

### 2.21. Cytotoxicity assay

The cytotoxicity of 2K4L was tested via the CCK-8 assay (Beyotime, Shanghai, China). Briefly, PMA-induced adherative THP-1 cells (5 × 10^5^/mL) were incubated with 2K4L (1.56 to 100 μM) in 96-well plates for 24 h. Under a dark condition, CCK8 solution was added to each well and cultured for 3 h. The optical density (OD) of each well at 450 nm was measured using a microtiter plate reader (Varioskan Flash Microplate Reader, Thermo Scientific Co., Beijing, China). Two duplicates were performed for each condition.

### 2.22. Mouse infection model

One hundred twenty wild-type male C57BL/6 mice (6–8 weeks old, weighing 18–20 g) were randomly divided into eight groups (*n* = 15/group): (1) 0.9% NaCl group (blank control); (2) MRAB 0227-infected group (control); (3) MRAB 0227 + dexamethasone (DXM, positive control); (4)–(5): MRAB 0227 + 2K4L (1 and 2 mg/kg weight, respectively); (6) AB 22933-infected group (control); (7) AB 22933 + DXM (positive control); and (8) AB 22933 + 2K4L (2 mg/kg weight). The mouse infection models were established as follows: briefly, mid-log phase of either MRAB 0227 (8 × 10^6^ CFU/mouse) or AB 22933 (1 × 10^8^ CFU/mouse) was injected i.p., into mice. After 24 h of infection, the animals received a daily treatment with 2K4L at different doses or 0.9% NaCl or DXM (2 mg/kg weight) for 7 days. Mouse stimulation reactions, the strength of activity, mental state, and body weight were recorded every day. Successful establishment of the model was marked by gradual development of some symptoms of sepsis in mice (no appetite, slow activity, erect body hair, unresponsive stimulation, sticky eye secretions, sticky stools, etc.). On Days 2 and 7 of peptide administration, the mice were sacrificed, the serum was collected for later analysis of liver functions and proinflammatory factors, and lung and liver tissues were collected for histochemical observation and Western blotting. Additionally, the liver, lung, kidney and blood on days 7 of peptide administration were collected for determination of bacterial density.

### 2.23. Liver function assay

The activities of alanine transaminase (ALT) and aspartate aminotransferase (AST) in mouse serum were determined to examine the magnitude of hepatic injury by using a VITROS 5600 automatic biochemical immunoassay analyzer (Ortho-Clinical Diagnostics, Inc.).

### 2.24. Histochemistry observation by H&E

At Days 2 and 7 of drug treatment, mouse livers, lungs and kidneys were collected and rinsed three times with normal saline and then immediately fixed in 4% formaldehyde before embedding in paraffin. Paraffin sections were sliced and stained with hematoxylin and eosin (H&E). The pathological changes in mouse tissue sections were observed under a microscope and photographed.

### 2.25. Bacterial colony counting in mouse organs

At Days 7 of peptide treatment, the mouse organs of livers, lungs, and kidneys with the mass of 1 g were collected and homogenized under sterile conditions. Homogenate was serially diluted in sterile saline, and spread on nutrient agar plates. The agar plates were incubated at 37°C overnight, and the bacterial colony in livers, lungs and kidneys (log10 CFU/g organ) of each mouse was counted. A similar method was performed to determine the CFU per mL of blood and the bacterial colony of blood (log10 CFU/mL blood) in each mouse was calculated. Two duplicates were performed for each condition.

### 2.26. Measurement of proinflammatory cytokines

The serum was collected from mouse blood on Days 2 and 7 of peptide administration to measure the proinflammatory factors TNF-α and IL-6. Proinflammatory cytokines were measured by using enzyme-linked immunosorbent assay (ELISA) kits (Neobioscience Technology, Shanghai, China) according to the manufacturer’s instructions. Three duplicates were performed for each condition.

### 2.27. Western blot

Proteins were extracted from mouse liver tissue using RIPA lysis buffer that was suitably combined with a mixture of protease inhibitors and phosphatase inhibitors. Protein concentrations in the supernatant were quantified by using a BCA Protein Assay Kit (Beyotime, Shanghai, China). Proteins were separated by 10% sodium dodecyl sulfate-polyacrylamide gel electrophoresis (SDS-PAGE) and then wet electrotransferred to polyvinylidene fluoride (PVDF) transfer membranes for 90 min. Then, 5% skim milk in TBST (20 mM Tris pH 7.5, 150 mM NaCl, and 0.1% Tween 20) was used to block the PVDF membranes at room temperature for 2 h. Subsequently, the membranes were incubated with primary antibodies against ERK, JNK, p38, I-κB and NF-κB and phosphorylated ERK, JNK, p38, I-κB and NF-κB overnight at 4°C. After washing with TBST, the membranes were incubated with horseradish peroxidase-conjugated secondary antibodies (1:5,000) for 2 h at room temperature. Finally, an ECL chemiluminescence substrate (Thermo Scientific, USA) was used to develop the immunoreactivity of the membranes. Protein-antibody complexes were detected using an Azure c500 imaging system (Azure Biosystems, USA) and normalized using GAPDH as an internal control. Two duplicates were performed for each condition.

### 2.28. Statistical analysis

All experiments were performed in triplicate. The results are generally expressed as the means and standard errors. Statistical analyses were performed by GraphPad Prism 6.0 software. A two-tailed unpaired Student’s *t*-test was used to test for statistical significance. Significance is indicated as * for *p* < 0.05 and ^**^ for *p* < 0.01.

## 3. Results

### 3.1. Antimicrobial activities of 2K4L against *A. baumannii*

**FIGURE 1:**
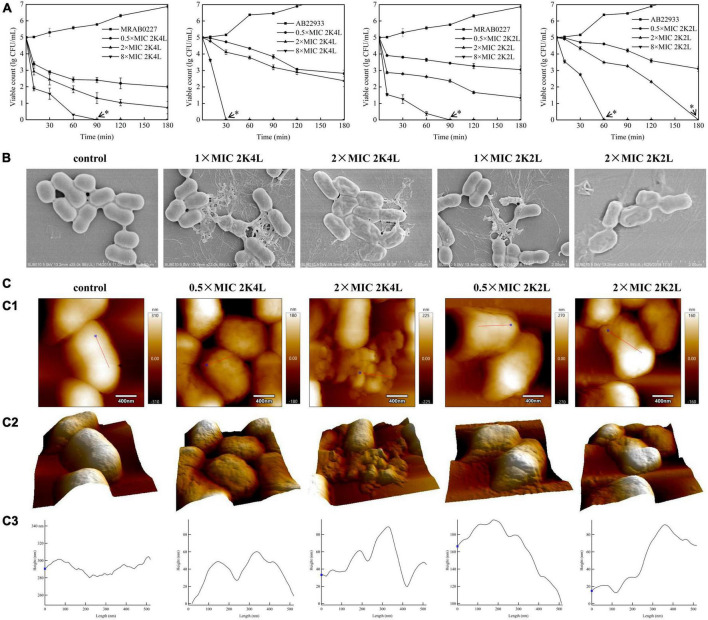
Bactericidal activity of the peptides against *A. baumannii*. **(A)** Killing kinetics of the peptides for the MRAB 0227 and AB 22933 strain. Co-incubation *A. baumannii* (1 × 10^5^ CFU/mL) with the different concentrations of peptides (0.5 × MIC, 2 × MIC and 8 × MIC) for 0–180 min. Aliquots were collected at 0, 15, 30, 60, 90, 120 and 180 min to count the bacteria. The data are presented as the mean ± SEM (*n* = 3). * in the figure suggested that no bacteria was detected at this concentration and time; **(B)** scanning electron micrograph of MRAB 0227 cells (B1) and MRAB 0227 (1 × 10^5^ CFU/mL) treated with 1 × MIC (B2) and 2 × MIC (B3) of 2K4L, 1 × MIC (B4) and 2 × MIC (B5) of 2K2L for 1 h (scale bar = 2 μm, *n* = 3); **(C)** atomic force microscope of MRAB 0227 cells (1 × 10^5^ CFU/mL) treated without (control) or in the presence of peptide for 1 h. An analysis of contact mode images was presented. **(C-1)** topography and **(C-2)** 3D images; **(C-3)** section profiles corresponding to red lines in **(C-1)** (scale bar = 400 nm, *n* = 3).

2K4L displayed strong antibacterial activity against *A. baumannii* with MIC values of 6.25 μM for MRAB 0227 and 3.13 μM for AB 22933. 2K2L had lower antibacterial activity than 2K4L, with MIC values of 25 μM for MRAB 0227 and 12.5 μM for AB 22933 (Ji et al., 2022). Compared to 2K2L, 2K4L has the same net positive charges (+5) but a high mean hydrophobic value (14.94 and 17.14, respectively), suggesting that the hydrophobicity of the peptide plays an important role in antibacterial activity against both multidrug-resistant and sensitive *A. baumannii*. The positive control Polymyxin B (PMB) and Gentamycin (GEN) displayed high antibacterial activity against MRAB 0227 and AB 22933. MIC values of PMB were 1.56 μM against MRAB 0227 and AB 22933, and GEN were 3.13 and 1.56 μM against MRAB 0227 and AB 22933, respectively. Piperacillin (PIP), a negative control had no antibacterial activity against MRAB 0227 and AB 22933 (MIC > 100 μM). The killing kinetics of the peptides against *A. baumannii* were shown in Figure 1A. The bactericidal activities of the peptides were time- and concentration dependent. The 2K4L at a concentration of 8 × MIC completely killed MRAB 0227 within 90 min but completely killed AB 22933 within only 30 min. The 2K2L at a concentration of 8 × MIC required 90 min and 60 min to completely kill MRAB 0227 and AB 22933.

The bactericidal function of the peptides on MRAB 0227 could be observed by SEM through the morphological changes of bacterial cells treated with the peptide for 1 h (Figure 1B). In the control group, untreated bacterial cells exhibited smooth and bright membrane surfaces. Treatment with 2K4L at concentrations of 1 × MIC and 2 × MIC led to rupture and collapse of bacterial cells and leakage of the intracellular contents. 2K2L at concentrations of 1 × MIC and 2 × MIC showed similar results to 2K4L.

The morphological changes of MRAB 0227 cells were further observed by using AFM (Figure 1C). The untreated MRAB 0227 cells were smooth surfaced and short rod shaped with a height of approximate 280 nm. After exposure to the peptides, the bacterial surface was highly granular with irregular grooves, and the bacterial collapse to a height of less than 100 nm, suggesting the release of bacterial intracellular contents. The irregularities were well reflected by the profiles presented in Figures C-1, C-2. The bacterial cells treated with 0.5 × MIC 2K4L exhibited deep grooves (20 nm deep and about 150 nm in diameter) in the central part of the cell, and the deep grooves were 80 nm as 2K4L concentration increased by 2 × MIC. While the bacterial cells treated with 2K2L at a concentration of 0.5 × MIC and 2 × MIC were observed deep grooves with 20 nm deep and about 100 nm in diameter. The analysis of cell surface roughness also confirmed the above results. The root-mean-square (RMS) roughness values of the control group were estimated as 14.63, and the RMS roughness values increased by 27.04 and 32.31 after the treatment of 0.5 × MIC and 2 × MIC of 2K4L, respectively. These results indicated that 2K4L and 2K2L damaged the integrity of MRAB 0227 cells, cause leakage of the intracellular contents, leading to a bacterial death. 2K4L showed stronger antibacterial activity than 2K2L against *A. baumannii* in a concentration-dependent manner (*p* < 0.01).

### 3.2. Membrane-activity mechanism of 2K4L against *A. baumannii*

#### 3.2.1. 2K4L damages membrane integrity and leads to the forming of membrane pore in *A. baumannii*

**FIGURE 2:**
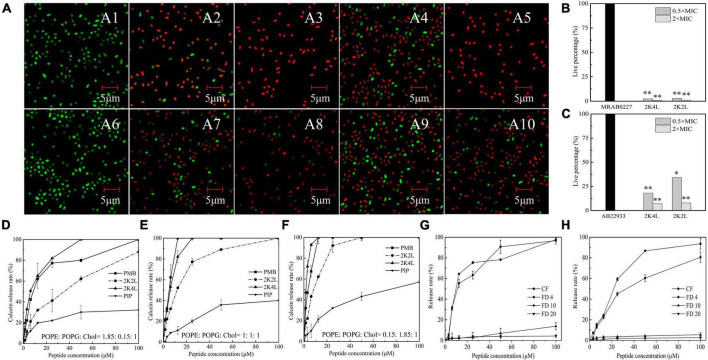
The effect of the peptides on bacterial inner membrane integrity and the leakage of fluorescein. **(A–C)** Laser scanning confocal microscopy images against *A. baumannii*. Mid-log phase AB 22933 and MRAB 0227 (1 × 10^5^ CFU/mL) were treated with peptides at a final concentration of 0.5 × MIC and 2 × MIC at 37°C for 1 h. The bacterial cells were washed, fixed, and stained with SYTO-9 (green) and PI (red). **(A1)** MRAB 0227 cells; **(A2,A3)** MRAB 0227 cells treated with 0.5 × MIC and 2 × MIC of 2K4L; **(A4,A5)** MRAB 0227 cells treated with 0.5 × MIC and 2 × MIC of 2K2L; **(A6)** AB 22933 cells; **(A7,A8)** AB 22933 cells treated with 0.5 × MIC and 2 × MIC of 2K4L; **(A9,A10)** AB 22933 cells treated with 0.5 × MIC and 2 × MIC of 2K2L (scale bar = 5 μm, *n* = 3); **(B,C)** live percentage of MRAB 0227 **(B)** and AB 22933 **(C)** cells treated with the peptide for 1 h. * (*P* < 0.05) and ^**^ (*P* < 0.01) indicate statistically significant differences between peptide and the MRAB 0227/AB 22933 group; **(D–F)** calceins release from LUVs liposomes composed of POPE: POPG: Chol = 1.85: 0.15: 1 **(D)**, POPE: POPG: Chol = 1: 1: 1 **(E)** and POPE: POPG: Chol = 0.15: 1.85: 1 **(F)**; **(G,H)** fluorescein release encapsulated in liposomes composed of PE: PG: CL = 18: 4: 3 by peptides [**(G)** 2K4L; **(H)** 2K2L)].

First, cell membrane damage to *A. baumannii* was observed by confocal laser scanning microscopy using a Live/Dead BacLight™ kit. SYTO-9 dye can penetrate intact cell membranes to show green fluorescence, and PI shows red fluorescence after entering bacterial cells through damaged cell membranes. Figure 2A illustrated an image of 100% green fluorescence (live bacteria) in the untreated MRAB 0227 (Figure 2A1) and AB 22933 (Figure 2A6) groups, while green fluorescence decreased, and red fluorescence occurred after treatment with 2K4L and 2K2L (Figures 2A2–A5, A6–A10). Treatment with 2K4L at concentrations of 0.5 × MIC and 2 × MIC decreased the percentages of live MRAB 0227 bacteria by 97.6 and 99.3%, respectively (Figure 2B), and killed AB 22933 cells by 81.9 and 93.0%, respectively (Figure 2C). 2K2L at a concentration of 2 × MIC decreased the percentages of live MRAB 0227 and AB 22933 bacteria by 96.0 and 92.2%, respectively (Figures 2B, C). The results suggested that 2K4L had a strong ability to disrupt the bacterial membrane system.

The membrane-disrupting activity of 2K4L as a cationic AMP on neutral and negatively charged membranes was investigated in three calcein-encapsulating large unilamellar vesicles (LUVs). The results showed that the peptides could rapidly disrupt the mimic membrane, resulting in leakage calceins in a concentration-dependent manner. As shown in Figures 2D–F, a treatment of 12.5 μM 2K4L caused 65.2, 82.3 and 100% calceins leakage from the LUVs at ratios of POPE/POPG/Chol = 1.85/0.15/1, 1/1/1 and 0.15/1.85/1, respectively, suggesting that 2K4L as a cationic AMP showed a strong ability to damage the negatively charged membrane. Similarly, 12.5 μM 2K2L caused 32.1, 52.2 and 62.3% calceins leakage from the LUVs at ratios of POPE/POPG/Chol = 1.85/0.15/1, 1/1/1 and 0.15/1.85/1, respectively. The 2K4L induced the release of calceins from LUVs with different compositions better than 2K2L (*P* < 0.05). The results suggested that 2K4L disrupted the LUVs membrane, resulting in leakage of the intracellular content (calceins), and the higher the negatively charged POPG proportion in the liposome membrane was, the greater the calcein release induced by the peptides.

Subsequently, the degree damage of bacterial membrane was determined by measuring the pore sizes of membrane using fluorescent dyes of various molecular weights and sizes in LUVs mimicking the cell membrane of *A. baumannii*. The diameter of the carboxyfluorescein (CF) was less than 1 nm and FITC-dextran 4 (FD 4), FITC-dextran 10 (FD 10) and FITC-dextran 20 (FD 20) was 2.8 nm, 4.6 nm and 6.6 nm, respectively. As shown in Figure 2G, FD 10 and FD 20 with diameters of 4.6 nm and 6.6 nm almost had not been released after a treatment of 2K4L for 15 min. While 2K4L at a concentration of 50 μM induced 90 and 80% leakage of CF and FD 4 with diameters of less than 2.8 nm, respectively, and the leakage of CF and FD 4 was more than 95% when 2K4L concentration increased 100 μM. 2K2L at a concentration of 50 μM induced 85 and 60% leakage of CF and FD 4, respectively, and 100 μM 2K2L lead to the leakage of CF and FD 4 was approximately 93 and 80%, respectively (Figure 2H). However, FD 10 and FD 20 almost had not been released after a treatment of 2K4L at a concentration of 100 μM for 15 min, suggesting that the pore size of LUVs mimicking the cell membranes of *A. baumannii* induced by 2K4L and 2K2L was less than 4.6 nm.

#### 3.2.2. 2K4L depolarizes the inner membrane and inserts into the membrane of *A. baumannii*

**FIGURE 3:**
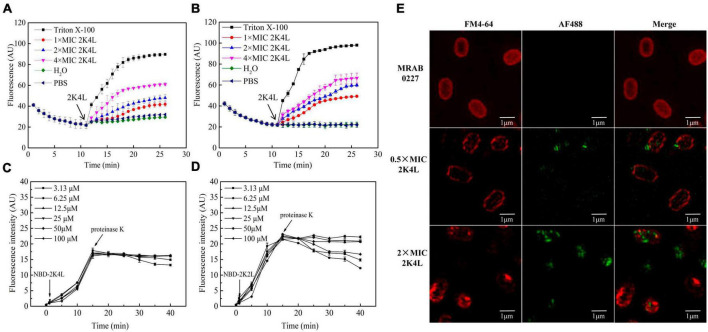
The inner membrane permeability of *A. baumannii* and accessibility of 2K4L to *A. baumannii*. **(A,B)** Depolarization of the inner membrane permeability of MRAB 0227 **(A)** and AB 22933 strain **(B)**. The bacterial suspension (1 × 10^5^ CFU/mL) was incubated with DiSC3-5 for 11 min, then peptides at final concentrations of 1, 2 and 4 × MIC were added to the bacterial samples. The data are presented as the mean ± SEM (*n* = 3); **(C,D)** protection of NBD-labeled 2K4L (3.13–100 μM) at various concentrations against proteolytic digestion in the presence of MRAB 0227 **(C)** and AB 22933 **(D)**. The data are presented as the mean ± SEM (*n* = 3); **(E)** super-resolution fluorescence microscopy images of the membrane of MRAB 0227 treated with 2K4L. The MRAB 0227 cell membrane was stained with FM4-64FX (red) and 2K4L with AF488 (green) in all images (scale bar = 1 μm, *n* = 3).

The inner membrane potential of *A. baumannii* was detected by using the membrane potential-sensitive dye DiSC3-5 to understand the depolarization ability of 2K4L on the *A. baumannii* inner membrane. As shown in Figures 3A, B, the fluorescence intensity of DiSC3-5 decreased as the dye concentrated in the cytoplasmic membrane from 0 to 11 min, resulting in a self-quenched fluorescence after the dye was added to the MRAB 0227 (Figure 3A) and AB 22933 (Figure 3B) cells. 2K4L was added to the bacterial samples until DiSC3-5 dye was taken up at a maximum amount at 11 min. 2K4L disrupted the membrane potential and caused cytoplasmic membrane depolarization of *A. baumannii* in a concentration-dependent manner, and complete membrane depolarization induced by the peptide occurred in 7–10 min. 2K4L at a concentration of 4 × MIC rapidly increased the fluorescence intensity of MRAB 0227 and AB 22933 cells by 68.0 and 68.1%, respectively, compared to the positive control Triton X-100 (defined as 100% cytoplasmic membrane depolarization).

The interaction mode between 2K4L and the membrane was investigated by measuring the fluorescence intensity of NBD-labeled 2K4L in the presence of liposomes. NBD fluorescence of NBD-labeled peptide on membrane surface can be degraded by protease K, leading to decrease of fluorescence intensity (Sun et al., 2015). The peptides could be hydrolyzed by proteases resulting in a decrease of fluorescence intensity. When peptides inserted into the membrane, NBD will not be hydrolyzed by proteinase K, and their fluorescence intensity will be sustained. As shown in Figure 3C, the fluorescence intensity of NBD-labeled 2K4L at 6.25–100 μM remained essentially unchanged after an addition of proteinase K, suggesting that the peptide was able to insert into the membrane. Figure 3D showed that the fluorescence intensity of NBD-labeled 2K2L at low concentration (3.13–12.5 μM) gradually reduced following the adding of protease K, but the fluorescence intensity was unchanged at 25–100 μM 2K2L. It was indicated that at low concentrations 2K2L remained on the bacterial membrane surface and susceptible to proteolytic attack by proteinase K, and high concentrations of 2K2L could be inserted into the membrane.

The effect of 2K4L on the bacterial membrane was observed by using super-resolution fluorescence microscopy imaging using 3D structured illumination microscopy (3D-SIM). FM4-64 is a membrane-selective red fluorescent dye used to label bacterial cell membranes and AF488 is a green fluorescence dye with amino-reactive activity to be used to label the peptide. Figure 3E showed the images of FM4-64FX-labeled lipid membranes of MRAB 0227 cells incubated with AF488-labeled 2K4L at a concentration of 0.5 × MIC and 2 × MIC for 1 h. After treatment with AF488-labeled 2K4L at a concentration of 0.5 × MIC for 1 h, 2K4L (green fluorescence) was localized uniformly around the MRAB 0227 cell membrane and observed on the membrane surface of the bacterial cells, but the membrane integrity was not damaged. However, when the concentration of 2K4L increased to 2 × MIC, MRAB 0227 cells were observed an incomplete membrane compared to the control group, suggesting that the high concentration of peptide disrupted the membrane integrity of MRAB 0227 cells. 2K2L exhibited similar damaging effects on the membrane structure integrity of MRAB 0227 cells (Supplementary Figure 2).

#### 3.2.3. Effect of 2K4L on the outer membrane of *A. baumannii*

**FIGURE 4:**
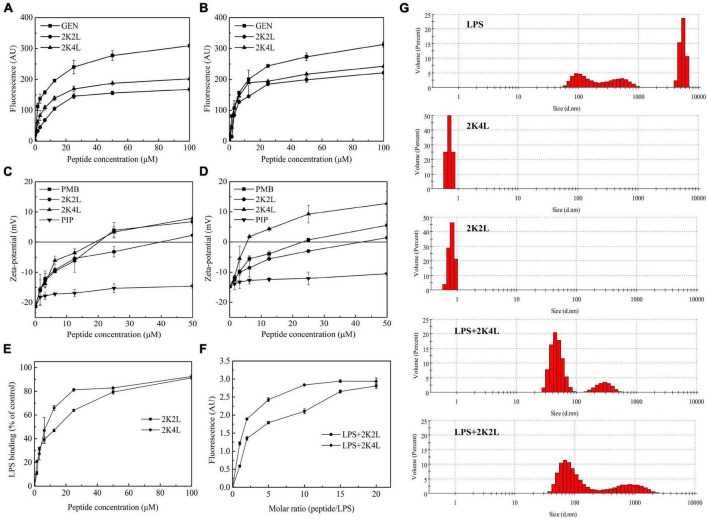
The outer membrane permeability of *A. baumannii* and interaction/disaggregation of LPS by the peptides. **(A,B)** The outer membrane permeability by NPN fluorescence detection of MRAB 0227 **(A)** and AB 22933 **(B)** after a treatment of the peptides. The data are presented as the mean ± SEM (*n* = 3); **(C,D)** zeta potential changes of MRAB 0227 **(C)** and AB 22933 **(D)** treated with the peptides. The data are presented as the mean ± SEM (*n* = 3); **(E)** the ability of the peptides to bind LPS using pierce™ chromogenic endotoxin quant kit. The data are presented as the mean ± SEM (*n* = 3); **(F)** fluorescence intensity changes of FITC-labeled LPS in the presence of the peptides; **(G)** size distribution of LPS, the peptides and LPS in the presence of the peptides by measure the particle size using DLS. The data are presented as the mean ± SEM (*n* = 3).

The permeability of the bacterial outer membrane was assessed by using the hydrophobic fluorescent probe 1-N-phenylnaphthylamine (NPN). NPN fluorescence is quenched in aqueous solution, whereas a strong fluorescence intensity is exhibited when NPN is exposed to a hydrophobic environment. Once the bacterial outer membrane is damaged, NPN enters the membrane, resulting in an increase in fluorescence intensity. The 2K4L induced a significant increase in the fluorescence intensity of NPN in MRAB 0227 (Figure 4A) and AB 22933 (Figure 4B) cells in a concentration-dependent manner. Compared with gentamicin (positive control), 12.5 μM 2K4L increased the fluorescence intensity by 70.7 and 94.4% in MRAB 0227 and AB 22933, respectively, and 12.5 μM 2K2L caused an increase in the fluorescence intensity by 53.7 and 72.4%, respectively. Both 2K4L and 2K2L were able to permeate into the hydrophobic outer membrane of *A. baumannii*, and 2K4L with high hydrophobicity exhibited a higher permeability ability than 2K2L (*P* < 0.05).

The zeta potential results indicated the interaction of the positively charged 2K4L and the negatively charged outer membrane surface of *A. baumannii*. After titration with 2K4L at concentrations ranging from 1.56to 50 μM, the net charges on the outer membrane surface changed from −21.4 to +7.9 mV for MRAB 0227 cells (Figure 4C) and from −14.8 to +12.8 mV for AB 22933 cells (Figure 4D), indicated that the negative charges on the outer membrane surface of *A. baumannii* cells were neutralized by the positive charges of the peptide. The results suggested that 2K4L could bind to the surface of *A. baumannii* cells through electrostatic interactions.

LPS is a major component of the outer membrane of Gram-negative bacteria, which serve as barrier for bacteria. Firstly, the interaction of the peptides and LPS was measured through the ability to neutralize LPS using a Pierce’s Endotoxin Quantitative Kit. As shown in Figure 4E, the peptides inhibited the LPS-mediated activation of the LAL enzyme. 2K4L inhibited more than 80% of the endotoxin at a concentration of 50 μM, while 2K2L did at a concentration of 100 μM. The result suggested that 2K4L and 2K2L could neutralize LPS.

LPS aggregates play a key role in protecting bacteria from invasion exogenous chemicals (Bello et al., 2015). Here, FITC- labeled LPS polymer depolymerization experiment was used to investigate the effect of 2K4L on the structure of LPS aggregates. Monomer-LPS molecules tend to aggregate with each other to form macromolecular aggregates with molecular weight of more than 1,000 KDa, but the fluorescence quenching occurred when FITC- labeled LPS formed soluble aggregates (Bhunia et al., 2009). As shown in Figure 4F, the fluorescence intensity gradually increased after the addition of the peptides. When the molar ratio of 2K4L/LPS increased to 10, the fluorescence intensity remained constant at 2.9 AU. The fluorescence intensity was stable at the molar ratio of 2K2L/LPS = 15. The results suggests that 2K4L and 2K2L can dissociate the aggregated state of LPS oligomers.

As shown in Figure 4G, LPS aggregates into two different sized polymers in its natural state, including a mean diameter of 100 nm (small, 48%) and 6,000 nm (large, 52%), respectively. However, in the presence of 2K4L, large LPS aggregates (a mean diameters of 6,000 nm) was totally disappeared indicating that 2K4L had a strong depolymerization on LPS aggregates. Similarly, 2K2L could disaggregate LPS aggregates.

By testing the antibacterial activity of 2K4L on protoplasts, it was demonstrated that the protection of the outer cell membrane against bacteria was the key to the activity of the antimicrobial peptide. The protoplast of *A. baumannii* was prepared by removing the bacterial cell wall with lysozyme. Compared with intact bacteria, 2K4L showed significantly increased antimicrobial activity against *A. baumannii* protoplasts. The MIC value of 2K4L against MRAB 0227 decreased fourfold from 6.25 to 1.56 μM, while the value against AB 22933 decreased fourfold from 3.13 to 0.78 μM. This result indicated that the outer membrane acted as a barrier to protect bacterial cells and affected the antibacterial activity of 2K4L against *A. baumannii*.

### 3.3. 2K4L inhibited the biofilm formation of *A. baumannii*

**FIGURE 5:**
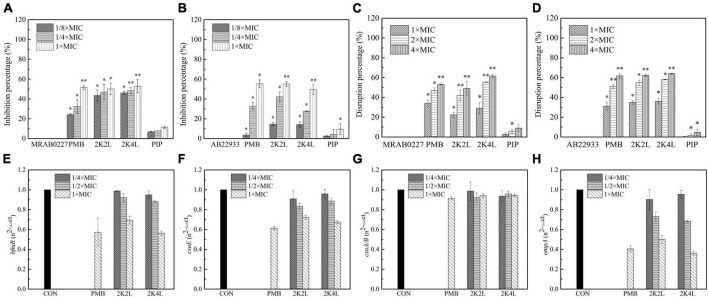
Effects of the peptides on *A. baumannii* biofilm. **(A,B)** Inhibitory percentage of the peptides (1/8 × , 1/4 × and 1 × MIC) on biofilm forming of MRAB 0227 and AB 22933 (1 × 10^5^ CFU/mL); **(C,D)** disaggregation of 1-day mature biofilm by the peptides (1 × MIC, 2 × MIC, and 4 × MIC); * (*P* < 0.05) and ^**^ (*P* < 0.01) indicate statistically significant differences between peptide and the MRAB 0227/AB 22933 group; **(E–H)** expression of biofilm associated genes *bfmR* **(E)**, *csuE* **(F)**, *csuA/B* **(G)** and *ompA* **(H)** of MRAB 0227 by RT-qPCR. The data are presented as the mean ± SEM (*n* = 3).

*A. baumannii* is prone to form biofilms on solid surfaces and cause infection in clinical settings (Sivaranjani et al., 2018). Here, we found that 2K4L exhibited not only membrane disruption activity against *A. baumannii* but also antibiofilm effects. The peptides inhibited biofilm formation in a concentration-dependent manner. Compared to the control (no peptide treatment), the biofilm formation was inhibited by 2K4L at concentrations of 1/8, 1/4 and 1 × MIC for 46, 48 and 53% in MRAB 0227 cells (Figure 5A), respectively. While the inhibition rate of 2K4L on the biofilm formation was for 14, 27 and 49% in AB 22933 cells (Figure 5B), respectively. The MBIC_50_ values of 2K4L on the MRAB 0227 and AB 22933 strains were approximately equal to the respective 1 × MIC value. The 2K2L and polymyxin B (PMB, a positive control) showed antibiofilm-forming effects similar to the antibiofilm-forming effects of 2K4L, but piperacillin (PIP, a negative control) did not show antibiofilm activity.

It was found that AMPs was less sensitive to the mature biofilms (Otto, 2006). Cationic peptides could be rejected or sequestered by the extracellular polymeric molecules of biofilms, significantly hindering the interaction with bacterial cells (Grassi et al., 2017). The result indicated that the 2K4L also had a disaggregation effect on the biofilm that had been formed for one day. The 2K4L at a concentration of 1 × MIC reduced the attached biofilm biomass by 29% for MRAB 0227 (Figure 5C) and by only 36% for AB 22933 (Figure 5D). The 2K2L at a concentration of 1 × MIC reduced the attached biofilm biomass by 22% for MRAB 0227 and by 35% for AB 22933. The results indicated that the peptides at a low concentration exhibited a low disaggregation effect on the 1-day mature biofilms of *A. baumannii*. As the peptide concentration increased to two to fourfold of the MIC, the attached 1-day mature biofilm biomass significantly decreased, but the survival rates of bacteria were found to be significantly decreased (Supplementary Figure 3), suggesting that 2K4L and 2K2L at a concentration greater than 2 × MIC might reduce the biomass of an attached 1-day mature biofilm by killing bacteria within it. The MBRC_50_ values of 2K4L and 2K2L on MRAB 0227 biofilm were approximately twofold and fourfold of the MIC and approximately twofold of the MIC for 2K2L and 2K4L on AB 22933.

Biofilm formation of *A. baumannii* is affected by many factors (Sivaranjani et al., 2018). Among these factors, there are some genes related to the adhesion and formation of biofilms on abiotic and biotic surfaces. Here, the *bmfR*, *ompA*, *csuA/B*, and *csuE* genes associated with the formation of biofilms were investigated to understand the effects of 2K4L on biofilm formation of MRAB 0227 by quantitative RT-qPCR. The results showed that 2K4L and 2K2L at a sub-MIC concentration (1/4 × MIC and 1/2 × MIC) could not decrease the expression of the genes *bmfR* (Figure 5E) and *csuE* (Figure 5F), and the gene expression was slightly decreased after treatment with 1 × MIC of peptide. The 2K4L showed no effect on the expression of the *csuA/B* gene (Figure 5G) but significantly decreased the expression of the *ompA* gene (Figure 5H). *OmpA* protein is one of the major outer membrane proteins of gram-negative bacteria related to maintaining the structural integrity and stability of the outer membrane and plays an important role in bacterial adherence and biofilm formation. The 2K4L reduced RNA levels of *bfmR*, *csuE*, and *OmpA*, which is likely to reduce the formation of biofilms.

### 3.4. Protective action of 2K4L on sepsis mice infected with *A. baumannii*

**FIGURE 6:**
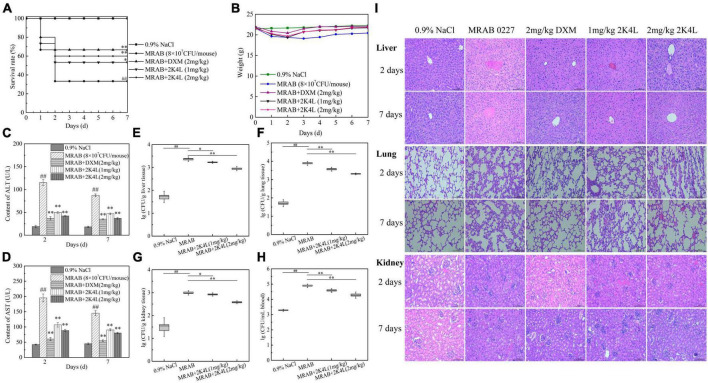
Protection of 2K4L against sepsis mice caused by the MRAB 0227 infection. A total of 6–8 weeks male C57BL/6 mice were injected with MRAB 0227 (8 × 10^6^ CFU/mouse) or AB 22933 (1 × 10^8^ CFU/mouse) and treated with 2K4L (1 and 2 mg/kg weight) or 0.9% NaCl or DXM (2 mg/kg weight) for 7 days (*n* = 15/group). On Days 2 and 7 of peptide administration, the serum was collected for liver functions and proinflammatory factors, and lung and liver tissues were collected for histochemical observation and Western blotting. The data are presented as the mean ± SEM (*n* = 3). **(A)** Survival rate of mice; **(B)** body weight of mice; **(C,D)** liver function (**C:** ALT; **D:** AST) analysis; **(E–H)** bacterial density in infected mouse organs. At Day 7 of peptide treatment, the liver **(F)**, lung **(G)**, kidney **(H)** and blood **(I)** of each mouse were collected and the bacterial density determined (*n* = 3). ^##^*P* < 0.01 indicates statistically significant differences between the MRAB 0227 group and 0.9% NaCl. *P < 0.05 and **P < 0.01 indicate statistically significant differences between peptide and the MRAB 0227 group; **(I)** pathological observation of liver, lung and kidney by H&E staining (scale bar = 200 m, *n* = 3).

2K4L had strong antibacterial activity against bacteria, but low toxicity in THP-1 cells. As shown in Supplementary Figure 4A, the survival rate of THP-1 cells gradually decreased with increasing concentration after co-incubation of 2K4L for 24 h. The concentration of 2K4L was less than 6.25 μM with a survival rate of more than 60%. As shown in Supplementary Figure 4B, the survival rate of 2K4L in mice (from 1 to 10 mg/kg body weight) was 100%. It was suggested that 2K4L exhibited non-toxic *in vivo*. To investigate the protective effects of 2K4L, mice were infected with *A. baumannii* and treated with the peptide after infection. After 2 days of injection of MRAB 0227 at a dose of 8 × 10^6^ CFU/mouse, the survival rate was only 33% of the 0.9% NaCl group (Figure 6A). The administration of 1 mg/kg and 2 mg/kg 2K4L increased the mouse survival rate to 53 and 60% of the 0.9% NaCl group, respectively. At 2 and 7 days after the administration of 2 mg/kg 2K4L, the mouse body weight significantly increased compared to the body weight of the MRAB 0227 infected mice (Figure 6B) (*p* < 0.001). The positive control DXM group had a 67% increased survival rate compared with the infected group. The infectious mouse model was also successfully established by injecting the sensitive *A. baumannii* AB 22933 strain. Treatment with 2 mg/kg 2K4L increased the mouse survival rate from 27% of the infected group to 60% (Supplementary Figure 3A). The body weight changes within 7 days of the administration of 2 mg/kg 2K4L were similar to the body weight changes of the MRAB 0227-infected group (Supplementary Figure 5B).

The liver function data showed that the ALT and AST levels of the MRAB 0227-infected model mice were significantly increased, and both were more than sixfold the levels of the control group, while the administration of 2K4L significantly decreased ALT and AST levels in a concentration-dependent manner (Figures 6C, D). At 7 days of administration of 2 mg/kg 2K4L, ALT and AST levels decreased by 57.6 and 44.6% compared to the MRAB 0227-infected model mice, respectively. In AB 22933-infected model mice, 2 mg/kg 2K4L decreased ALT and AST levels by 57.8 and 47.2%, respectively (Supplementary Figures 5C, D).

The antibacterial efficacy *in vivo* was examined in sepsis mice induced by *A. baumannii*. As shown in Figures 6E–H, MRAB 0227 infection significantly increased the bacterial density in the mouse organs compared with 0.9% NaCl group. On 7 days, the treatment of 2 mg/kg 2K4L significantly reduced the MRAB 0227 density in the liver (Figure 6E), lung (Figure 6F), kidney (Figure 6G) and blood (Figure 6H) by 61.4, 73.3, 61.1, and 75.3%, respectively. At 7 days of administration of 2 mg/kg 2K4L, the AB 22933 density in the liver (Supplementary Figure 5E), lung (Supplementary Figure 5F), kidney (Supplementary Figure 5G) and blood (Supplementary Figure 5H) by 88.7, 76.7, 61.1 and 56.1%, respectively.

The mouse liver, lung and kidney were collected and examined by histopathology. As shown in Figure 6I, the liver tissues were severely damaged, manifested as swollen, necrotic and irregular distribution of liver cells in MRAB 0227-infected model mice. The lung tissues showed alveolar defects or swelling, as alveolar septa were significantly thickened and inflammatory cells were increased in the alveoli, and some degree of tissue deformation was displayed in MRAB 0227-infected model mice. MRAB 0227 infection damaged mouse kidneys. The glomeruli showed morphological changes, and the capillaries in the glomeruli were dilated. The glomerular sacs shrank in space and even adhered to the glomeruli. At 2 days and 7 days after 2K4L administration, the damaged liver, lung and kidney were significantly improved. These results indicated that 2K4L could protect mouse organs from damage caused by *A. baumannii*.

**FIGURE 7:**
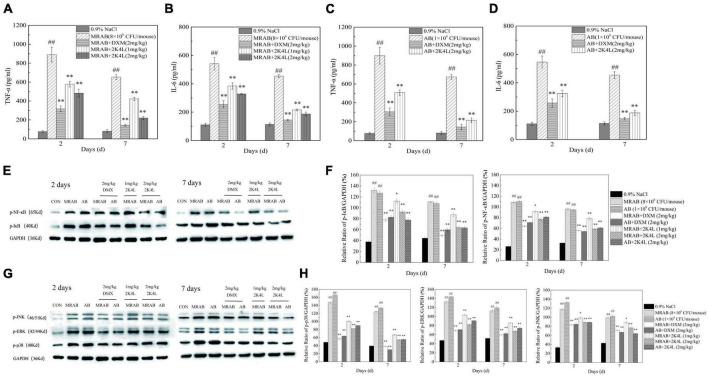
2K4L inhibited inflammatory response induced by *A. baumannii* in sepsis mice. **(A–D)** Effects of 2K4L on the production of TNF-α and IL-6 in the sepsis mice serum induced by MRAB 0227 and AB 22933; **(E,F)** proteins were extracted from mouse liver tissue at Days 2 and 7 of peptide treatment. Western blotting analysis of the effects of 2K4L on the expression of *p*-NF-κB and *p*-IκB; **(G,H)** western blotting analysis of the effects of 2K4L on the expression of *p*-JNK, *p*-ERK and *p*-p38. ^##^ (*P* < 0.01) indicates statistically significant differences between the MRAB 0227/AB 22933 group and control (0.9% NaCl). * (*P* < 0.05) and ^**^ (*P* < 0.01) indicate statistically significant differences between peptide and the MRAB 0227/AB 22933 group. The data are presented as the mean ± SEM (*n* = 3).

The anti-inflammatory effects of 2K4L were investigated in sepsis mice induced by *A. baumannii*. First, the levels of TNF-α and IL-6 in mice were detected. As shown in Figures 7A, B, the levels of TNF-α and IL-6 were significantly increased in MRAB 0227-treated sepsis mice. The treatment of 2 mg/kg 2K4L inhibited the production of TNF-α in mice infected with MRAB 0227 by 45.7 and 75.3% on days 2 and 7, respectively. Consistently, 2K4L 2 mg/kg treatment reduced IL-6 in mice infected with MRAB 0227 by 39.4 and 65.5% on days 2 and 7. In line with the above data, the release of TNF-α and IL-6 was reduced by 2K4L in AB 22933-treated sepsis mice (Figures 7C, D).

The NF-κB signaling pathway is a classic pathway that regulates inflammation (Lawrence, 2009). As shown in Figures 7E, F, the expression of phosphorylated IκB (*p*-IκB) and NF-κB p65 (*p*-p65) significantly increased in MRAB 0227- and AB 22933-induced sepsis mouse livers compared with the control group. After 2 days of 2K4L administration, the expression levels of *p*-IκB and *p*-p65 were downregulated by 29.8 and 28.8%, respectively. And the expression levels of *p*-IκB and *p*-p65 were downregulated by 42.4 and 38.9%, respectively, at 7 days compared to the MRAB 0227-treated sepsis mice. However, the expression levels of non-phosphorylated IκB and NF-κB p65 were different in the liver tissue of each administration group (Supplementary Figures 6A, B), suggesting that 2K4L reduced the inflammatory response by downregulating the phosphorylation levels of the signaling proteins IκB and NF-κB in *A. baumannii*-treated sepsis mice.

The mitogen activated protein kinase (MAPK) signaling pathway is another of the typical inflammatory response pathway (Leifer and Medvedev, 2016). Here, the phosphorylation of the signaling proteins in MAPK pathway were determined to further understand the anti-inflammatory effects of 2K4L. In addition, the effects of 2K4L on the MAPK signaling pathway were examined. As shown in Figures 7G, H, MRAB 0227 infection significantly increased the phosphorylated levels of p38 (*p*-p38), ERK (*p*-ERK) and JNK (*p*-JNK) in mouse livers. A total of 1 mg/kg 2K4L at 2 days downregulated the expression of *p*-p38 by 31.9%, *p*-ERK by 22.1% and *p*-JNK by 16.2% compared to the group of MRAB 0227-induced shock mice, respectively. While 2K4L at 7 days downregulated the expression of *p*-p38 by 54.7%, *p*-ERK by 35.1% and *p*-JNK by 25.1%, respectively. When the dose of 2K4L increased to 2 mg/kg, the expression levels of *p*-p38 were downregulated by 63.2%, *p*-ERK by 48.9% and *p*-JNK by 34.6% at 7 days of 2K4L treatment. The expression levels of non-phosphorylated p38, ERK and JNK were different in the liver tissue of each administration group (Supplementary Figures 6C, D). The results suggested that 2K4L downregulated the phosphorylation level of MAPK signaling proteins in *A. baumannii*-induced sepsis mice.

## 4. Discussion

*Acinetobacter baumannii* is one of the most troublesome pathogens causing various pathogenic infections in the modern healthcare system, since it is able to escape from available antibiotics resulting in multidrug-resistant strains (Peleg et al., 2008). The increasing antibiotic resistance of *A. baumannii* is related to its multiple drug resistance mechanisms, including enzymatic modification of antibiotic molecules, modification of antibiotic target sites, expression of efflux pumps, and downregulation of cell membrane porin channel expression (Anaya-López et al., 2013). The development and research of antimicrobial agents against multidrug-resistant *A. baumannii* is urgent, while antimicrobial peptides are a potentially effective option. In particular, the killing effect of cationic antimicrobial peptides on bacteria is rapid and efficient, directly acting on the bacterial cell membrane, so resistance is less likely to develop (He et al., 2021). The 2K4L is composed of 4 positively charged lysine residues and amidation of carboxyl terminals, which endows it with broad-spectrum antibacterial activity against gram-negative and gram-positive bacteria (Ji et al., 2022). In the present study, 2K4L, as a cationic peptide, displayed high antibacterial activity against multidrug-resistant *A. baumannii* (MRAB0227 strain) and sensitive *A. baumannii* (AB22394 strain), with MIC values of 6.25 and 3.13 μM, respectively. In addition, high hydrophobicity (17.14) and amphipathic α-helical confirmation in a membrane-mimetic environment enhanced the antibacterial potency of 2K4L against the *A. baumannii* strain. The 2K2L is an analog of 2K4L and is composed of the same net positive charge of 5 but low hydrophobicity (14.94) compared to 2K4L. Consequently, 2K4L showed a fourfold higher antibacterial potency against MRAB 0227 and AB 22394 than 2K2L. The present results further confirmed that hydrophobicity promoted the interaction of cationic AMPs with the more negatively charged bacterial cell membrane and increased antimicrobial potency (Hong et al., 2021). The 2K4L at high concentrations (8 × MIC) rapidly killed AB 22933 and MRAB 0227 only within 30 min and 90 min, suggesting that the peptide was clearly bactericidal against *A. baumannii* and similar to other frog skin peptides (das Neves et al., 2019; Sharma et al., 2019; Luo and Song, 2021). Scanning electron microscopy and atomic force microscope images confirmed that 2K4L resulted in bacterial cell rupture and collapse, leading to bacterial death.

AMPs, especially cationic AMPs, are universally accepted to act as completely different action mechanisms from traditional antibiotics (Ciumac et al., 2019; Tornesello et al., 2020; Juhl et al., 2021). According to previous literature, a two-step mechanism of AMPs is adopted: (i) they selectively bind to negatively charged bacterial surfaces by electrostatic interaction, causing the outer membrane permeability to change; and (ii) unstructured linear peptides in solution fold into an amphipathic α-helical conformation in the membrane environment, which is easy to insert into the cytoplasmic membrane, leading to leakage of cell contents because of transmembrane potential changes, finally resulting in cell death (Sinha et al., 2017; Bechinger et al., 2020). Similar to other cationic AMPs, 2K4L exhibited strong electrostatic interactions with the negatively charged outer membranes of *A. baumannii* and increased outer membrane permeability. Five net positive charges of 2K4L have been confirmed to facilitate interaction with MRAB 0227, and AB 22933 cell walls contain negatively charged lipopolysaccharides (zeta potential). The 2K2L with the same net positive charges (+5) showed similar effects on the outer membranes of MRAB 0227 and AB 22933 cells, suggesting that positive charges in AMPs play an important role in interacting with the bacterial outer membrane. While LPS exists as aggregates in the outer membrane of cells, FITC-labeled 2K4L fluorescence and DLS measurement also clearly demonstrated that 2K4L could dissociate the aggregated state of lipopolysaccharides. These results indicated that 2K4L interacted with the lipopolysaccharides head group through electrostatic interaction, which caused 2K4L to insert into the acyl-chain of lipopolysaccharides through hydrophobic interaction leading a significant perturbation of the lipopolysaccharides packing organization.

Although there is no universal agreement regarding the precise mechanism of AMPs to the cytoplasmic membrane, the carpet model is broadly accepted (Fernandez et al., 2012; Bechinger, 2015). In this model, AMP first deposits on the cell membrane surface like a carpet by binding its positive charges to the negatively charged phospholipid head groups and then inserts into the hydrophobic core of the lipid bilayer as the AMP concentration increases, destabilizing the cell membrane and eventually forming holes or pores to kill bacterial cells (Wang et al., 2016; Usmani et al., 2021). Our data showed that 2K4L could rapidly depolarize the cytoplasmic membrane of MRAB 0227 and AB 22933 in a concentration-dependent manner, leading to large-scale permeabilization of the membrane and eventually killing bacterial cells. In this process, the hydrophobicity of AMP is a main influential factor in the interaction with the bacterial cell membrane. NBD- labeled peptides fluorescence experiment suggested that 2K4L could insert into PE/PG/CL LUVs membrane. Due to the fluorescence intensity showing practically no change when proteinase K was added, it indicated that the NBD-terminal of 2K4L totally penetrated into the LUVs membrane within 3.13–100 μM. Value compared with 2K4L, 2K2L with a low hydrophobic which may be related to its limited ability inserted into the membrane at high concentration. Then 2K4L damaged the integrity of the bacterial cell membrane, resulting in the inflow of SYTO-9 and even PI dyes. Similar results were also observed in mimic membranes, and 2K4L led to the formation of a pore between 2.6 nm and 4.6 nm in mimic membranes of MRAB 0227.

Many reports have shown that biofilm formation is one of the major causes of the rapid development of *A. baumannii* resistance to antimicrobials (Irani et al., 2018; Shahed-Al-Mahmud et al., 2021). *A. baumannii* biofilms colonize a variety of surfaces, including healthcare-associated medical devices (Pompilio et al., 2021), hospital furniture (Orsinger-Jacobsen et al., 2013), and even most abiotic surfaces (Gaddy et al., 2009; Paricio et al., 2019), leading to an increase in the long-term survival of *A. baumannii* in the hospital environment and causing medical device-associated infections (Choi et al., 2009). The 2K4L at subinhibitory concentrations (1/8 and 1/4 × MIC) inhibited the biofilm formation of multidrug-resistant *A. baumannii* by approximately 50%, suggesting that 2K4L could interfere with the normal formation of biofilms at a concentration not enough to fully inhibit bacterial growth. The antibiofilm activity of 2K4L was also assessed in another stage: in 24 h mature biofilms. However, 2K4L at 1 × MIC concentrations only reduced the attached biofilm biomass by 30%, and the 24 h mature biofilms significantly reduced when the peptide concentration increased to two to fourfold of the MIC, but bacterial survival rates were found to be significantly decreased. The results suggested that the peptides at a concentration greater than 2 × MIC might decompose the formed biofilm by killing bacteria since bacterial biofilms are more difficult to eradicate than their planktonic counterparts.

Biofilm formation/maintenance of *A. baumannii* can be influenced by many factors, including nutrient availability (Flannery et al., 2020; Nie et al., 2020), macromolecular secretions (Luo et al., 2015; Moon et al., 2017) and complex regulatory networks, such as the chaperone-usher (Csu) pili system (Kim et al., 2019) and two-component system (*BfmS*/*BfmR*) (Kim et al., 2019). The production of pili and the formation of biofilms on abiotic surfaces depend on *csuE* gene expression of the csuA/BABCDE locus regulated by *bfmS* and *bfmR* genes in *A. baumannii*. However, our present study showed that 2K4L slightly decreased the gene expression of *bmfR* and *csuE* and had no effect on the expression of the *csuA/B* gene. Interestingly, 2K4L significantly decreased the expression of the *ompA* gene encoding *OmpA* protein (outer membrane protein A), a macromolecular secretion that plays an important role in *A. baumannii* pathogenesis, such as cell adherence, biofilm formation, antimicrobial resistance, serum resistance, apoptosis and immunomodulation (An et al., 2019; Tiku et al., 2021). *OmpA* from *A. baumannii* is believed to make up a potential virulence factor with multiple important effects in pathogenesis and signal processing (Kwon et al., 2017; Shirazi et al., 2019). The 2K4L has been proven to be able to reduce the expression of some biofilm-related genes, thereby inhibiting the adhesion and formation of biofilms.

Clinical application of AMPs is often limited by severe destability and toxicity *in vivo*. 2K4L was non-toxic to mice. To investigate the protective effects of 2K4L *in vivo*, we examined the function of 2K4L in a sepsis mouse model with *A. baumannii* infection. Indeed, direct evidence has shown that 2K4L significantly increased the survival rate of infected mice and decreased ALT and AST levels in serum compared to MRAB 0227-infected model mice. Additionally, 2K4L exhibited strong efficacy by reducing bacterial organ density in the mouse spesis model causing that severe damage of mouse liver, lung and kidney tissues caused by MRAB 0227 infection was improved. It indicated that the low toxicity of 2K4L was *in vivo* and had the protection of sepsis mice by reducing bacterial burden. The present study also demonstrated that 2K4L could reduce the release of TNF-α and IL-6 showing significant anti-inflammatory activity *in vivo*, and downregulate the phosphorylation levels of MAPK and NF-κB signaling proteins in the proinflammatory pathway activated by *A. baumannii* infection. The results suggested that 2K4L not only exerted antibacterial activity against multidrug-resistant *A. baumannii* and multidrug-sensitive *A. baumannii in vitro* but also protected sepsis mice *in vivo*.

In conclusion, 2K4L presented high antibacterial potential against *A. baumannii in vivo* and *in vitro*, especially killing a clinically isolated multidrug-resistant strain by targeting the non-receptor-mediated membrane system and biofilm formation. Most importantly, 2K4L showed remarkable protection in *A. baumannii*-infected sepsis mice by downregulating the expression of proinflammatory signaling pathway proteins, leading to a decrease in the production of the proinflammatory factors TNF-α and IL-6. Therefore, 2K4L can be expected to be a potential candidate to fight increasing bacterial resistance and can be developed as a new therapeutic agent for *A. baumannii*-induced sepsis.

## Data availability statement

The original contributions presented in this study are included in this article/Supplementary material, further inquiries can be directed to the corresponding author.

## Ethics statement

The animal study was approved by the Liaoning Normal University. The study was conducted in accordance with the local legislation and institutional requirements.

## Author contributions

FaJ: Data curation, Formal analysis, Methodology, Writing – original draft, Writing – review and editing. GT: Data curation, Writing – review and editing. DS: Data curation, Funding acquisition, Methodology, Writing – original draft, Writing – review and editing. FeJ: Writing – review and editing.
