# Supplementary figures and images for: Antimicrobial peptide 2K4L disrupts the membrane of multidrug-resistant Acinetobacter baumannii and protects mice against sepsis
Source: Front Microbiol. 2023 Oct 24;14:1258469. doi: 10.3389/fmicb.2023.1258469 (PMC10628664; doi:10.3389/fmicb.2023.1258469)

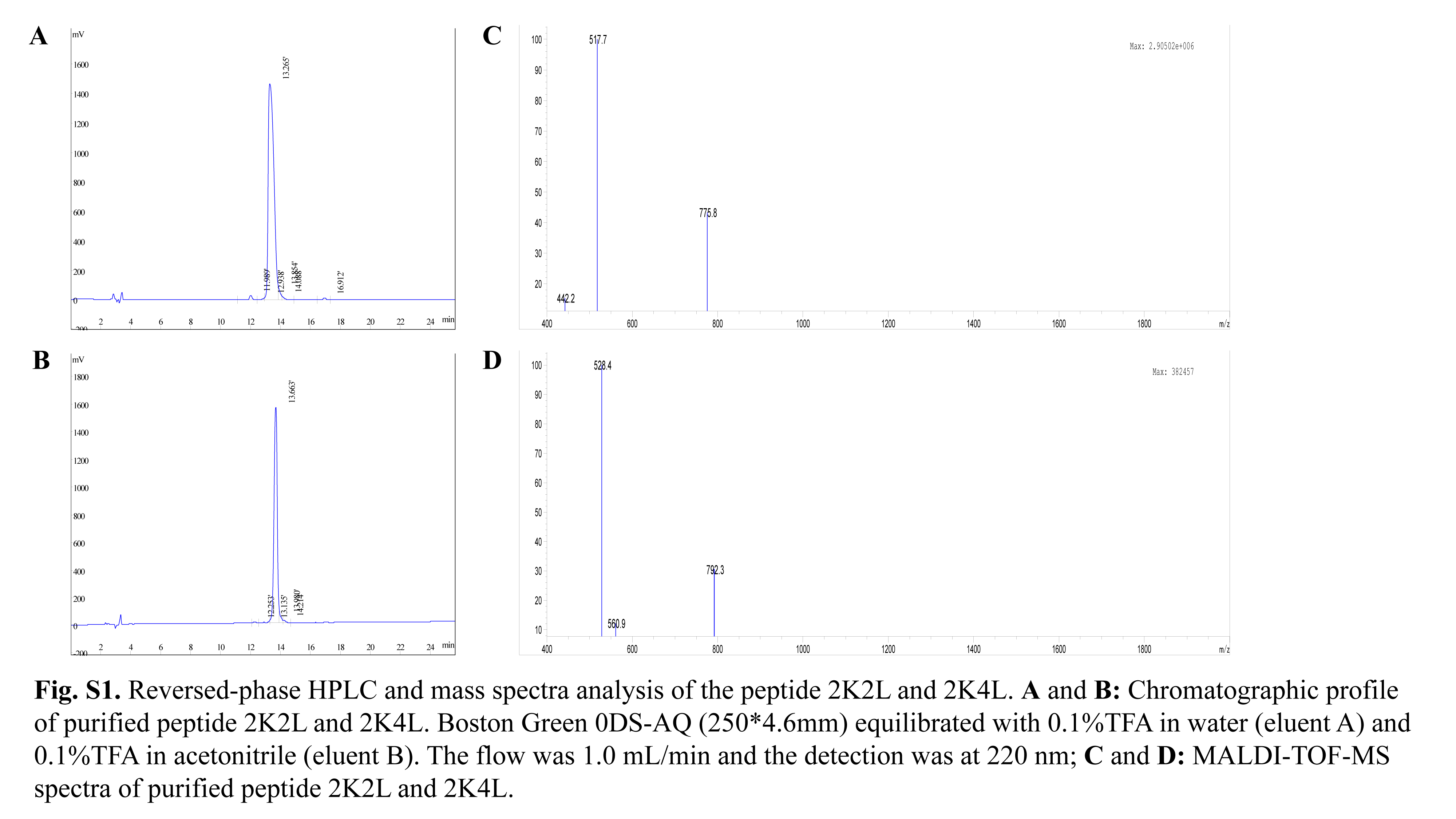

Supplement: Supplementary file 1 [file Image_1.jpg]

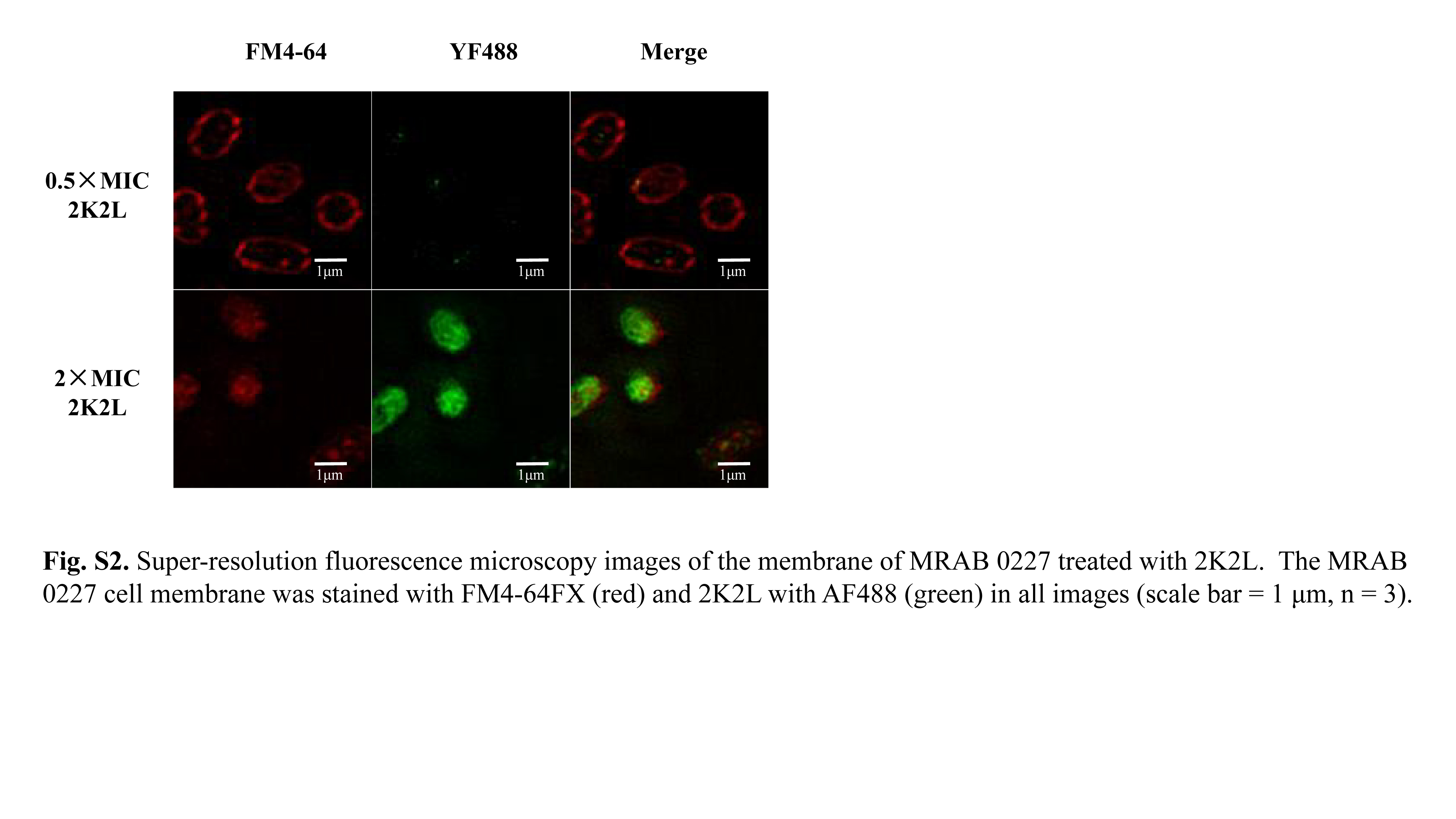

Supplement: Supplementary file 2 [file Image_2.jpg]

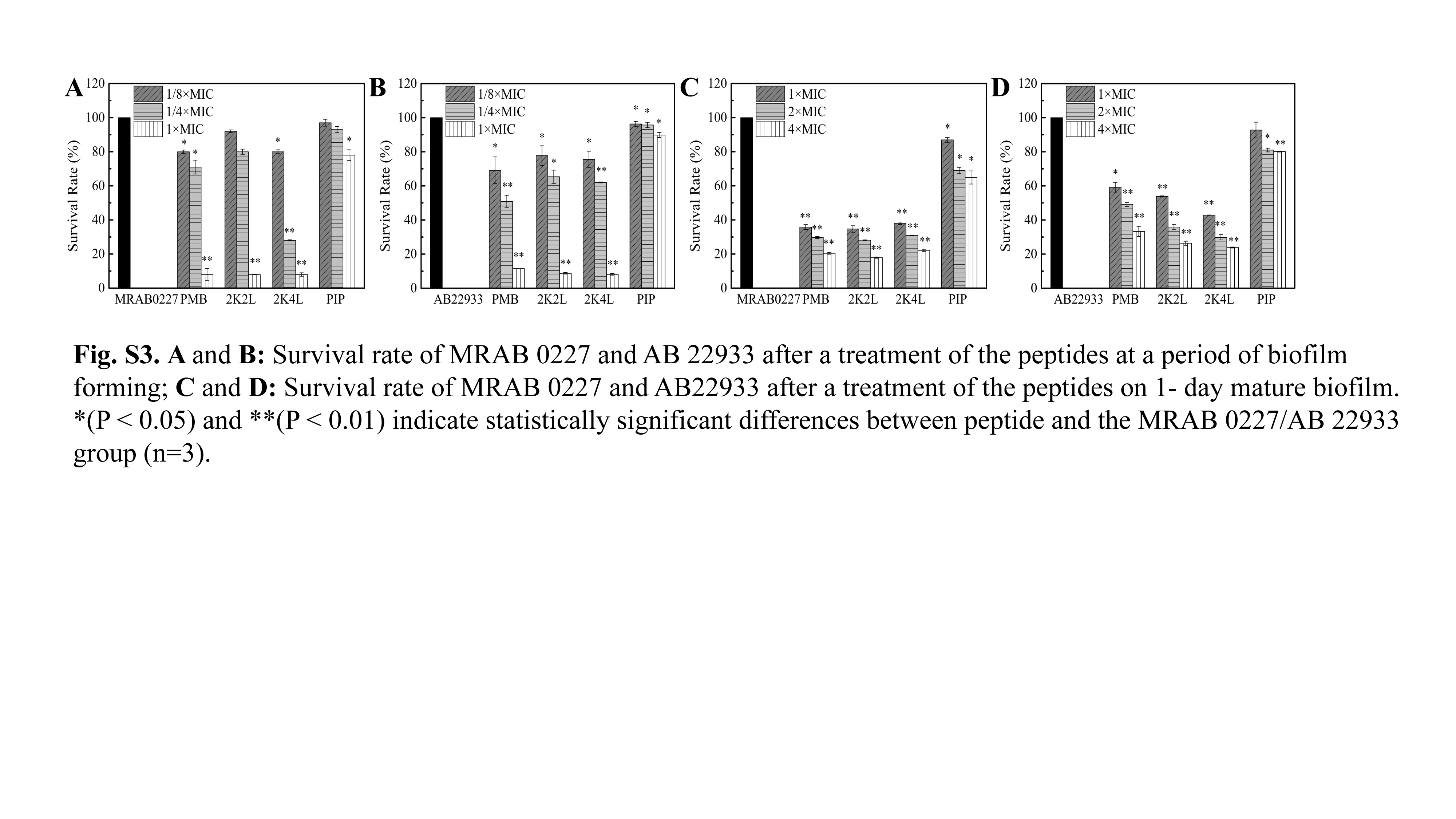

Supplement: Supplementary file 3 [file Image_3.jpg]

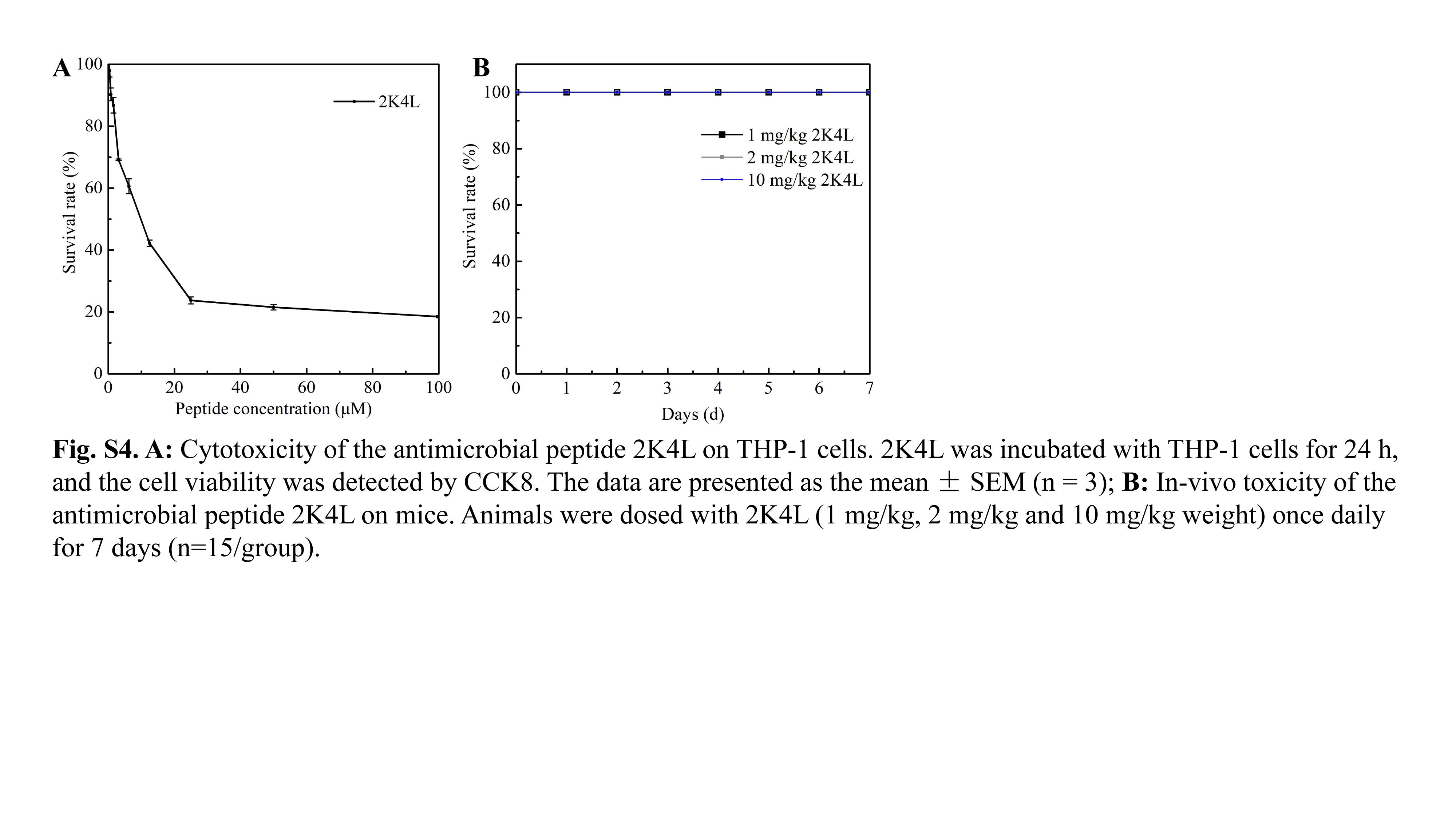

Supplement: Supplementary file 4 [file Image_4.jpg]

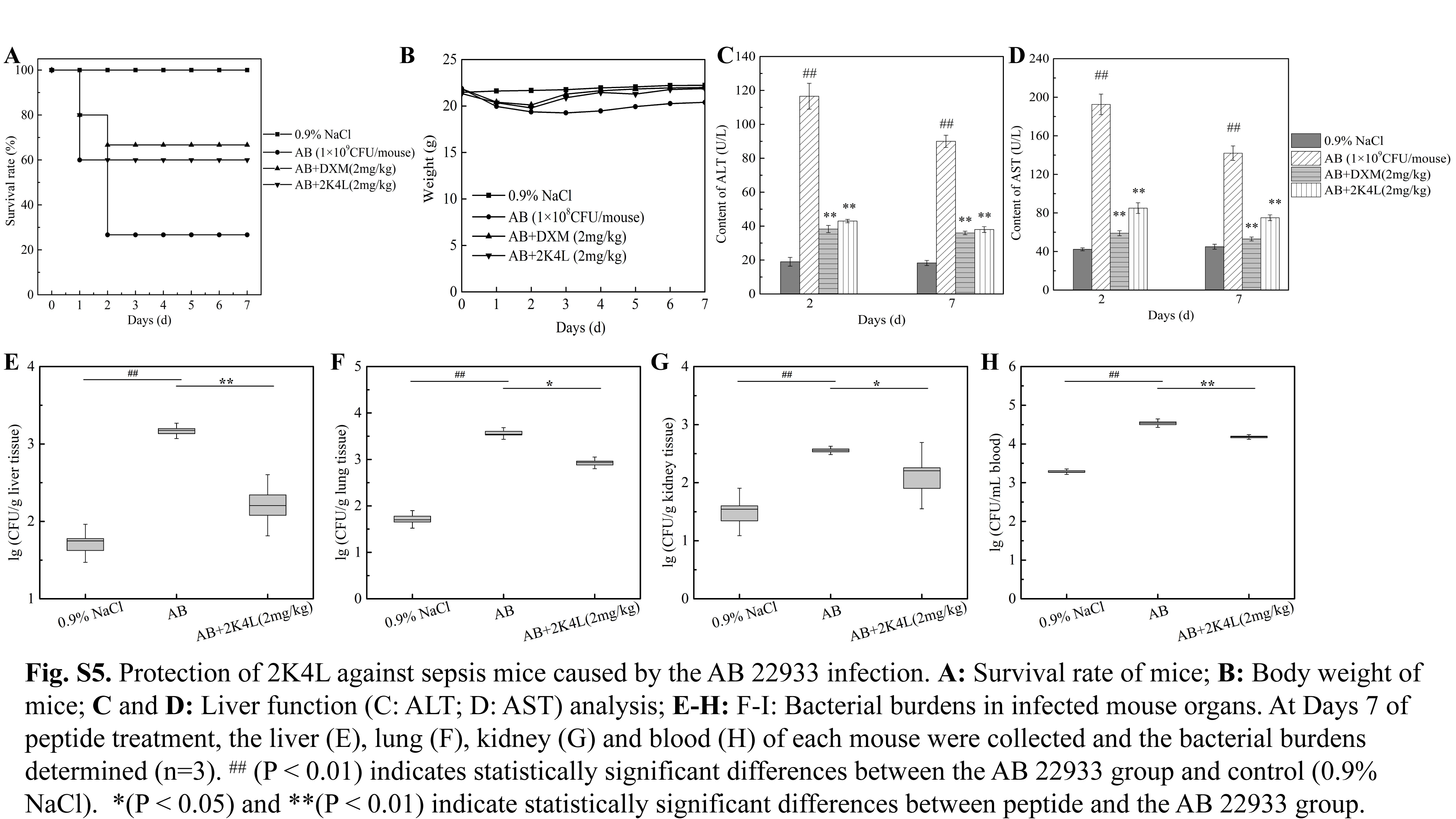

Supplement: Supplementary file 5 [file Image_5.jpg]

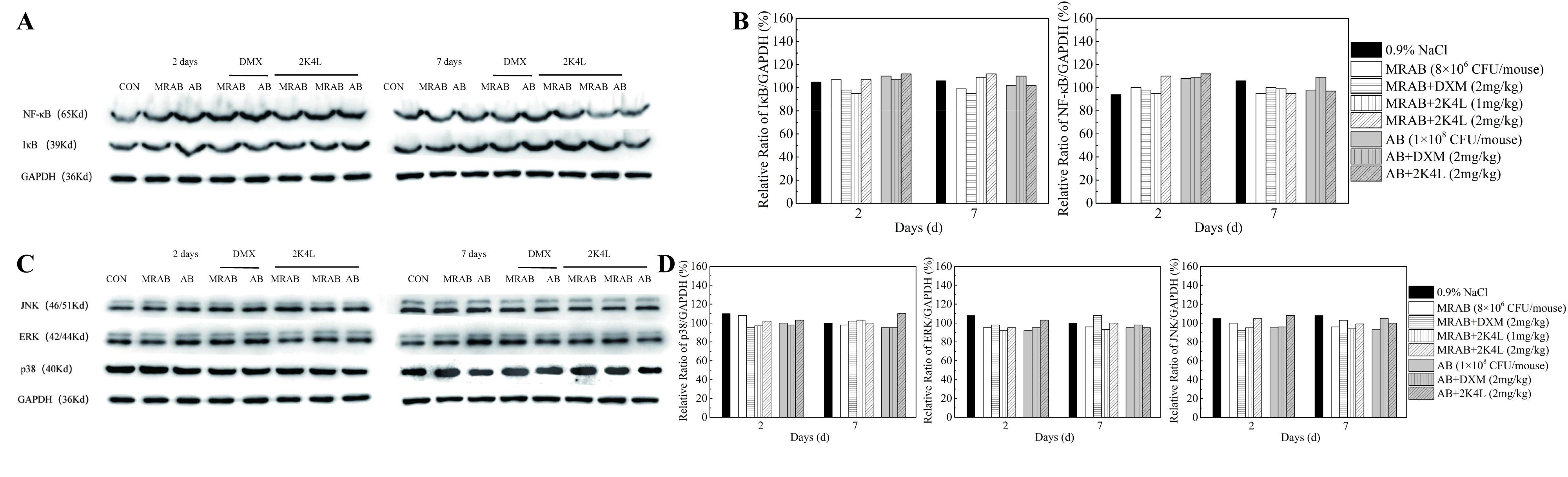

Supplement: Supplementary file 6 [file Image_6.JPEG]
